# Computational modeling of phagocyte transmigration for foreign body responses to subcutaneous biomaterial implants in mice

**DOI:** 10.1186/s12859-016-0947-3

**Published:** 2016-02-29

**Authors:** Mingon Kang, Liping Tang, Jean Gao

**Affiliations:** Department of Computer Science and Engineering, University of Texas at Arlington, 500 UTA Blvd., Arlington, 76019 USA; Department of Bioengineering, University of Texas at Arlington, 500 UTA Blvd., Arlington, 76019 USA

**Keywords:** Computational modeling, Foreign body responses, Phagocyte transmigration, Parameter estimation

## Abstract

**Background:**

Computational modeling and simulation play an important role in analyzing the behavior of complex biological systems in response to the implantation of biomedical devices. Quantitative computational modeling discloses the nature of foreign body responses. Such understanding will shed insight on the cause of foreign body responses, which will lead to improved biomaterial design and will reduce foreign body reactions. One of the major obstacles in computational modeling is to build a mathematical model that represents the biological system and to quantitatively define the model parameters.

**Results:**

In this paper, we considered quantitative inter connections and logical relationships among diverse proteins and cells, which have been reported in biological experiments and literature. Based on the established biological discovery, we have built a mathematical model while unveiling the key components that contribute to biomaterial-mediated inflammatory responses. For the parameter estimation of the mathematical model, we proposed a global optimization algorithm, called Discrete Selection Levenberg-Marquardt (DSLM). This is an extension of Levenberg-Marquardt (LM) algorithm which is a gradient-based local optimization algorithm. The proposed DSLM suggests a new approach for the selection of optimal parameters in the discrete space with fast computational convergence.

**Conclusions:**

The computational modeling not only provides critical clues to recognize current knowledge of fibrosis development but also enables the prediction of yet-to-be observed biological phenomena.

**Electronic supplementary material:**

The online version of this article (doi:10.1186/s12859-016-0947-3) contains supplementary material, which is available to authorized users.

## Background

Medical implants, such as breast implants, encapsulated tissues/cells, neural electrodes, and eye implants, have experienced remarkable development and growth during the past decades. In the meantime, an increasing number of medical implant failures also has been reported. In earlier studies, it is well documented that excessive fibrotic responses are responsible for the failure of many medical implants [1–4]. Medical implants provoke unpredicted responses and reactions of the immune system, which are fibrotic capsule formations surrounding the medical device. To be specific, wound healing responses start with acute inflammatory responses, and then follow with fibrotic tissue reactions. The continuous inflammatory responses lead to overwhelming fibrotic reactions. Hence, comprehensive understanding of the mechanism governing the reactions can play an important role in successful implantation and development of the biomaterial while reducing its side effects and simultaneously improving the functionality of the implants.

Computational modeling and simulation have been highlighted in biomedical research for decades, since they not only discover the nature and their associations of the biological components but also provide quantitative predictions that go beyond the biological experiment. Therefore, in-depth understanding of the foreign body responses and its computational modeling will disclose the contributing components and help to predict the evolution, which would eventually lead to reduced failure rate of implantation. A number of research has been conducted for modeling and predicting foreign body fibrotic reactions. A hybrid model was proposed by combining a differential equation system and kinetics Monte Carlo algorithm to simulate and predict phagocytes responses at molecular level [5]. Chemical kinetics equations were adapted for building a predictive tool of foreign body fibrotic reactions [6, 7]. Partial differential equations were also utilized for macrophage spatial/temporal dynamics in foreign body reactions[8]. 

Tang et al. had conducted biological experiments with mice to recognize major components involved in foreign body reactions and to discover their responses and reactions [2]. To summarize the current biological understanding, the evolution of biomaterial-mediate inflammatory responses may be divided into six consecutive events: (1) phagocyte transmigration through the endothelial barrier, (2) chemotaxis toward the implants, (3) adherence to the biomaterial, (4) phagocyte activation, (5) fibrin deposition, and (6) fibroblast proliferation and collagen production [2]. Among these six procedures, we focused on comprehensive modeling of phagocyte transmigration. It is assumed that certain components such as mast cells, histamine, histamine receptors, and P/E selectins, are mainly involved in the event [9]. Histamine release has been reported to trigger inflammatory responses to biomaterial implants in human [10], and the event was reported by observing the concentration change of polymorphonuclear neutrophils (PMN) and monocytes/macrophages (M *Φ*), which are the most abundant type of phagocytes.

**Fig. 1:**
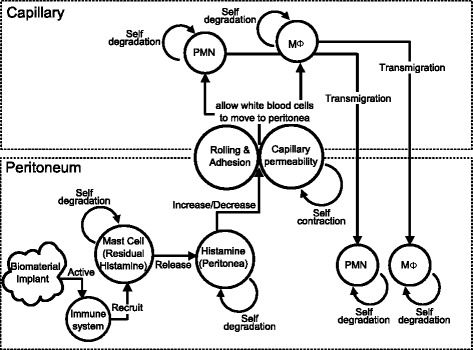
Overview of phagocyte transmigration

Phagocyte transmigration is known as one of the major reactions of the immune system. Different components contributing to the process are hypothesized based on our new biological experiments and reported discovery [2, 11]. Figure 1 describes the elements - mast cells, histamine, histamine receptors, and P/E selectins - involved in phagocyte transmigration and their interactive roles as a summary.

In this study, we conducted a mathematical modeling of phagocyte transmigration which is one of the processes involved in fibrosis formed around an implanted biomedical device. Furthermore, a new optimization approach, Discrete Selection Levenberg-Marquardt Algorithm (DSLM), based on our previous preliminary work [12] is developed for parameter estimation of the dynamic system for the accurate simulation of the biologic system. The new parameter estimation approach satisfies global non-linear optimization and converges in a feasible time. We will elucidate the biological experiment and elaborate the successive courses in the following sections.

## Methods

### Biological experiments for phagocyte transmigration

We have carried out biological experiments to build phagocytes transmigration models. We chose and tested the model system by using the experimental results obtained from mice, since a mouse model is commonly used to test the compatibility of medical implants and also to mimic foreign body reactions in human. For implantation, Swiss-Webster mice were anesthetized with isoflurane (Abbott Laboratories, North Chicago, IL), a 1.5 cm mid-abdominal or mid-dorsal longitudinal incision was made, and 0.6 cm diameter test disks and drug release pumps were implanted intraperitoneally (5 disks/mouse). The incision was closed with stainless steel wound clips. For studies in which the net recruitment of phagocytes was measured, control animals (operated but without implant placement) were always included. Immediately prior to examination, the animals were euthanized with *CO*_2_. The implants were carefully removed from the peritoneal cavity or subcutaneous space and washed with sterile PBS. These samples were used for assay of inflammatory and fibrotic responses.

Inflammatory cells are recruited to the interface between biomaterial implants and skin tissue, which is the area of interest in this study. For measurements of the acute inflammatory responses, investigation was typically performed after sixteen hours. The sixteen hour time point is the earliest time point for maximal inflammatory cell recruitment.

**Fig. 2:**
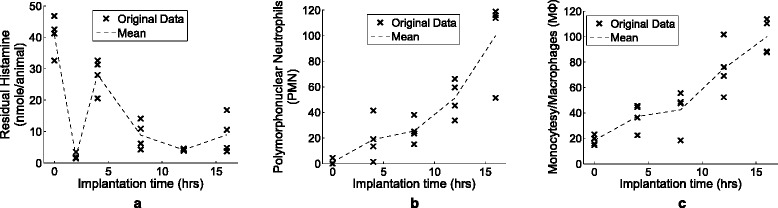
Experimental measurements acquired from Swiss-Webster mice of same age shipment batch. **a** residual histamine, (**b**) polymorphonuclear neutrophils (PMN), and (**c**) monocytes/macrophages (M *Φ*). A ‘x’ represents a measurement of a mouse at the designed time, and dotted-lines show the evolutionary development of the cells on average

We measured the residual histamine release amount at 0, 2, 4, 8, 12, 16 hours (Fig. 2a), and PMN and M *Φ* at 0, 4, 8, 12, 16 hours (Fig. 2b, c), where four mice were used for each time point. Residual histamine is granules in mast cells that are about to be released as histamine. Since histamine has a very short half-life (seconds to minutes) and is hard to measure reliably, the degree of histamine is approximately estimated by residual histamine. Note that PMN and M *Φ* were measured as the most abundant type of phagocytes.

**Fig. 3:**
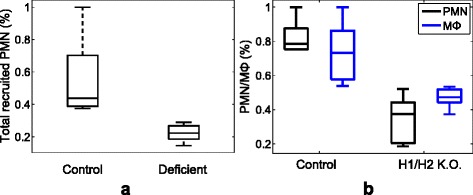
Experimental measurements for the deficiency study. **a** the accumulation of PMN on the control and mast cell knockout mice (5 mice for each, % of control) and (**b**) total recruited PMN and M *Φ* with H1/H2 receptor knockout (6 mice for each, % of control). The box spans the standard deviation of the data and its central mark is the median. The edges of the box shows the 25th and 75th percentiles of the data

For a knockout experiment, PET disks were implanted in the peritoneal space for two groups of mice - normal mice and mast cell-deficient mice. Mast cells are known as the major source of histamine. The PET disk activate a large amount of mast cells in the peritoneal space which in return releases the largest amount of histamine and consequently recruits phagocytes. It was observed that the number of totally recruited phagocytes surrounding implants was significantly decreased for mast cells deficient mice comparing to normal mice (Fig. 3a). For a normal mice, after implantation, phagocytes were recruited from the capillary to the peritoneal space where the implant was located, whereas the cell-deficient mice produce only half phagocytes comparing to the normal mice.

Previous studies hypothesized that histamine might also play an important role in the recruitment of inflammatory cells to implants. To verify the idea, histamine receptors, antagonist pyrilamine (H1 receptor antagonist) and famotidine (H2 receptor antagonist), were injected to the peritoneal space. The combined treatment of H1 and H2 receptor antagonists dramatically decreased the number of phagocytes on implant surfaces as well as the number of PMN and M *Φ* recruited *de novo* to the peritoneal cavity (Fig. 3b). The other intracellular substances via complex intracellular signaling may interfere with the activity of the inhibitory and stimulating actions, but the potential receptor interactions and signaling pathway have yet to be investigated. Therefore, in this study, we assume that histamine enhances phagocyte transmigration via both H1 and H2 receptors.

The half of the phagocytes in the knockout experiments (Fig. 3) can be suspected as a delayed accumulation. However, the knockout mice have *normal* inflammatory responses as shown in the previous work [13]. Moreover, the knockout mice triggered reduced long-term foreign body responses, fibrotic tissue formation, was shown in a recent publication [14].

## Modeling for phagocyte transmigration

In this section, we build computational models of phagocyte transmigration by utilizing reported biological literature and investigating the phenomena of the observations.

### Residual histamine

Residual histamine is granules in mast cells. In a biological sense, the phenomenon that residual histamine decreases up to the first two hours can be described as the residual histamine’s degranulation from mast cells for histamine releasing (Fig. 2a). In addition, according to the experimental observations and literature [15, 16], another legitimate assumption that the number of new mast cells is dramatically increased by the immune system shaped as a damped harmonic oscillation after two hours may be persuasive. Modeling biological systems with damped oscillation can be widely adapted in computational biology [17]. Therefore, the source of mast cells is modeled by:

(1) $$ \ddot{U}_{xrmc}(t) + \beta \dot{U}_{xrmc}(t) + {\omega_{0}^{2}}(t){U}_{xrmc}(t) = 0,  $$

where *U*_*xrmc*_ is the function of the source that releases new mast cells (mast cell source), *β* is a non-negative constant for resistance of friction and mass, and *ω*_0_(*t*) indicates the oscillator frequency. The explicit forms for (1) are:

(2) $$\begin{array}{@{}rcl@{}} U_{xrmc}(t) &=& A(t) + A(t) \exp(-{\beta} (t-t_{1}))  \\ && \cos(\sqrt{1 - {\beta}^{2}} \omega_{0}(t) (t - t_{1})), \\ A(t) &=& k_{e0} \exp(-k_{k1}(t - t_{1})), \\ \omega_{0}(t) &=& k_{w0} \exp(-k_{k2}(t-t_{1})),  \end{array} $$

where *k*_*e*0_ is the initial value of the source, *k*_*k*1_ is the contraction rate of the source, *k*_*w*0_ is the initial value of oscillation frequency, *k*_*k*2_ is the contraction rate of oscillation frequency, and *t*_1_ is the starting point that the source is released.

Most biological components can be represented by a half-life cycle. In quantity analysis, a system is comprised of propagation and self-degradation as conflicting contexts. In this sense, the concentration of residual histamine over time *C*_*rh*_(*t*) can be modeled by the following equation:

(3) $$ \dot{C}_{rh}(t) = - k_{rhch} C_{rh}(t) + U_{xrmc}(t),  $$

where *k*_*rhch*_ is the rate that residual histamine degrades.

### Histamine

Histamine *C*_*h*_(*t*) increases as much as residual histamine decreases:

(4) $$ \dot{C}_{h}(t) = k_{rhch} C_{rh}(t) I_{mc}(t) - k_{hs} C_{h}(t),  $$

where *k*_*rhch*_ is the histamine release rate which is the same as the residual histamine degrading rate as given in (3), and *k*_*hs*_ is the rate that histamine degrades itself. The first term on the right hand side of (4) represents the increase of histamine released from mast cells. *I*_*mc*_(*t*) indicates the relative concentration level of mast cells for the knock-out experiment using mast cell deficient mice, where 0 ≤*I*_*mc*_(*t*)≤ 1. The second term on the right hand side of (4) represents the degradation of histamine itself.

### H1/H2 Histamine receptor

Histamine receptors enhance the permeability of the endothelial cell barrier of the capillary for the phagocyte to transmigrate into the peritoneal space. Histamine exerts its action only if combined with histamine receptors. The action is determined by histamine receptors - H1 histamine receptor or H2 histamine receptor. Therefore, the concentration of meaningful histamine receptors *C*_*hr*_(*t*) can be determined by the degree of histamine:

(5) $$ \dot{C}_{hr}(t) = \frac{k_{hchrt} C_{h}(t)}{k_{hchrb} + C_{h}(t)} - k_{hrs} C_{hr}(t),  $$

where *k*_*hchrt*_ and *k*_*hchrb*_ are the upper/lower bounds of rate that histamine receptors combine with histamine, and *k*_*hrs*_ is the rate that histamine receptors degrade themselves. In the same way as histamine modeling, the first term on the right hand side represents the propagation of histamine receptors combined with histamine, while the second term shows the degradation rate of histamine receptors. However, we adapted a hyperbolic form for the first term in (5), where it sets a maximally increasable bound with the change of histamine receptors since it would not increase unlimitedly.

### P/E selectins

Selectin-mediated leukocyte binding and migration are parts of the leukocyte recruitment processes. P and E selectins control the phagocyte’s rolling and adhesion on endothelial cells of the capillary while histamine receptors prompt to increase permeability of the capillary [11]. As same as histamine receptors, P and E selectins *C*_*s*_(*t*) are stimulated by histamine, where histamine participates in the upregulation of the selectin on endothelium [11]. The mathematical equation for P and E selectins are formulated as:

(6) $$ \dot{C}_{s}(t) = \frac{k_{hcst} C_{h}(t)}{k_{hcsb} + C_{h}(t)} - k_{ss} C_{s}(t),  $$

where *k*_*hcsb*_ and *k*_*hcst*_ are the upper/lower bounds of rate released by histamine in a hyperbolic form, and *k*_*ss*_ is the selectins degradation rate.

### Phagocytes

PMN and M *Φ* are the most common phagocytes. The total recruited PMN and M *Φ* represent the concentrations of transmigrated phagocytes into the peritoneal space and surrounding implants. For the modeling of PMN and M *Φ*, we considered the capillary permeability for PMN and M *Φ*, *C*_*pmnp*_(*t*) and *C*_*mpp*_(*t*), that represent the transmigration rates of the phagocyte, and formulated them as:

(7) $$\begin{array}{@{}rcl@{}} \dot{C}_{pmnp}(t)& = &\frac{k_{pmnipt} C_{hr}(t) C_{s}(t) I_{pmns}(t) I_{pmnhr}(t)}{k_{pmnipb} + C_{hr}(t) C_{s}(t) I_{pmns}(t) I_{pmnhr}(t)}\\ && - k_{pmnps} C_{pmnp}(t),\\ \dot{C}_{mpp}(t)& = &\frac{k_{mpipt} C_{hr}(t) C_{s} I_{mps}(t) I_{mphr}(t)}{k_{mpipb} + C_{hr}(t) C_{s} I_{mps}(t) I_{mphr}(t)}\\ && - k_{mpps} C_{mpp}(t),  \end{array} $$

where *k*_*pmnipb*_, *k*_*pmnipt*_, *k*_*mpipb*_ and *k*_*mpipt*_ are the upper/lower bounds of rate for histamine receptors and selectins that increase permeability respectively, and *k*_*pmnps*_ and *k*_*mpps*_ are the contraction rate of the capillary permeability. *I*_*pmnhr*_(*t*) and *I*_*mphr*_(*t*) are the constants indicating block/non-block histamine receptors, and *I*_*pmns*_(*t*) and *I*_*mps*_(*t*) are the regulations that control block/non-block selectins.

Then, the total-recruited PMN and M *Φ*, *C*_*pmn*_(*t*) and *C*_*mp*_(*t*), are modeled as:

(8) $$\begin{array}{@{}rcl@{}} \dot{C}_{pmn}(t) &=& C_{pmnp}(t) - k_{pmns} C_{pmn}(t), \\ \dot{C}_{mp}(t) &=& C_{mpp}(t) - k_{mps} C_{mp}(t),  \end{array} $$

where *k*_*pmns*_ and *k*_*mps*_ are the rates that PMN and M *Φ* degrade.

## Results

### Parameter estimation and simulation for phagocyte transmigration

#### Parameter estimation for phagocyte transmigration

The above section introduced a dynamic system which contains numerous parameters whose values reflect characteristics of the system. Thus, estimating parameters of the system is essential to discover the components’ behavior in the system and hence provides a successful quantitative modeling.

**Fig. 4:**
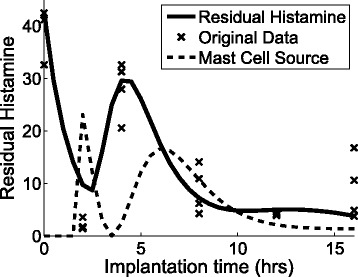
*In silico* estimation of residual histamine over time. The bold solid line, which is the *in silico* estimation, shows that the result is about the same as the synthetic data. The synthetic data is depicted with the mean (*thin solid line*) and the standard deviation (*bars*). The dotted line shows the estimated the mast cell sources which release new mast cells

**Fig. 5:**
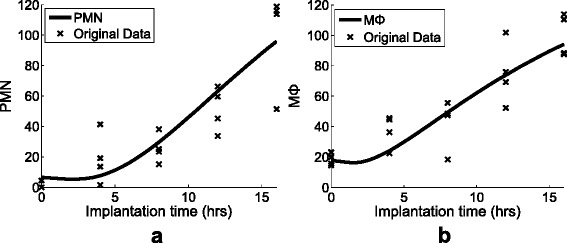
*In silico* estimation of (**a**) PMN and (**b**) M *Φ*

In this study, a total of 22 parameters for Eqs.(2)–(8) were estimated by performing Discrete Selection Levenberg-Marquardt (DSLM) with 100 synthetic data sets of every 30 minutes generated by Iterative Weighted Mean algorithm (IWM) and interpolation (see Model parameter estimation for DSLM and supplementary document for IWM). The optimally estimated parameters are listed in Table 1. The *in silicon* estimations for the residual histamine, PMN, and M *Φ* are depicted in Fig. 4 and Fig. 5, respectively. In Fig. 4, the bold solid line, which is the estimation of DSLM, shows that the result of residual histamine. The estimation appears to represent well the dynamic pattern that the residual histamine shows in the original observation (Fig. 4). The dotted line in Fig. 4 shows the estimated mast cell sources which release new mast cells. Subsequently, the computational models for the recruited phagocytes (PMN and M *Φ*) in the peritoneal space estimate closely to the biological observation in Fig. 5.

**Table 1:** Estimated parameters by DSLM

Parameter	Description	Estimation
*t*_1_	Starting time that the external source is released	3.5000
*k*_*e*0_	An initial concentration of the external source	11.2464
*k*_*k*1_	A self contraction of the external source	0.1017
*β*	An initial concentration of the oscillation bound	0.0134
*k*_*w*0_	An initial value of the oscillation frequency	1.4037
*k*_*k*2_	A contraction rate of the oscillation frequency	0.0937
*k*_*rhch*_	A rate that the residual histamine decayed	0.3704
*k*_*hs*_	A rate that the histamine regulates itself	0.0002
*k*_*hchrb*_	A rate that the histamine receptors are released	0.9997
*k*_*hchrt*_	A upper bound rate that the histamine receptors are released	0.0757
*k*_*hrs*_	A rate that the histamine receptors regulate themselves	0.7232
*k*_*hcsb*_	A lower bound of rate that the selectins are released	1.9997
*k*_*hcst*_	A upper bound of rate that the selectins are released	0.2668
*k*_*ss*_	A rate that the selectins regulate themselves	0.1024
*k*_*pmnipb*_	A lower bound of rate that increases the permeability for PMN	0.2226
*k*_*pmnipt*_	A upper bound of rate that increases the permeability for PMN	2.0000
*k*_*pmnps*_	A rate that the permeability of the capillary self degrades	0.1003
*k*_*mpipb*_	A lower bound of rate that increases the permeability for M *Φ*	0.0001
*k*_*mpipt*_	A upper bound of rate that increases the permeability for M *Φ*	1.0108
*k*_*mpps*_	A rate that the permeability of the capillary self degrades	0.1961
*k*_*pmns*_	A rate that the PMN self degrades	0.0582
*k*_*mps*_	A rate that the M *Φ* self degrades	0.0307

### Simulation for phagocyte transmigration

**Fig. 6:**
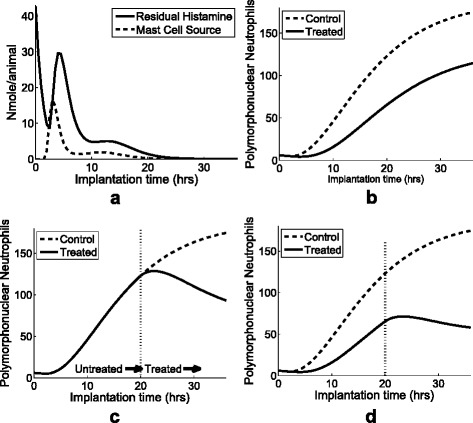
Simulation of residual histamin, PMN, and M *Φ*. **a** *In silico* estimation of residual histamine (*solid*) and mast cell source (*dotted*) up to 36 hours. The simulation prediction of recruited PMN while (**b**) mast cells are treated, (**c**) H1/H2 histamine receptors are treated after 20 hours, and (**d**) mast cells are blocked before first 20 hours and H1/H2 histamine receptors are treated after 20 hours. The dotted lines of (**b**-**d**) describe the simulation result under normal condition

One of the important goals in biological system modeling is to predict the unknown and yet to be biologically observed or a validated phenomenon. Although our lab experiment data only lasted up to 16 hours, we can go beyond that by using the computational model. Figure 6a shows the *in silico* estimation of the dynamic system for both residual histamine and mast cell source up to 36 hours. To predict how the system behaves under different perturbations, we observed the prediction of recruited PMN while blocking mast cells between 0 and 36 hours (Fig. 6b), blocking H1/H2 histamine receptors after 20 hours (Fig. 6c), and blocking mast cells for the first 20 hours and H1/H2 histamine receptors after 20 hours (Fig. 6d). The simulation performances were illustrated as solid lines comparing to the prediction of the control as dotted lines in Fig. 6b–d. From the *in silico* estimations in Fig. 6, we can predict the unobserved progression of the phagocyte: (b) PMN recruits about half amount of the control when mast cells are treated, (c) PMN decreases dramatically after blocking H1/H2 histamine receptors after 20 hours, and (d) PMN increases slowly with mast cell knockout for the first 20 hours and decreases with H1/H2 histamine receptors knockout after 20 hours. The tool to predict the progress of the phagocyte might play an important role in reducing the medical implant failure due to the excessive fibrotic responses.

## Model parameter estimation

The parameters of the proposed mathematical equations can be estimated by nonlinear programming algorithm. There are a number of numerical optimization techniques such as Newton’s method, Broyden’s method, line search method, and trust-region method in nonlinear programming. Those methodologies are all local optimization techniques, where the object function is globally convex. However, the object function of the proposed models is unfortunately non-convex, which needs a global optimization technique. Therefore, in this study, we developed a global optimization algorithm, called Discrete Selection Levenberg-Marquardt (DSLM) for the parameter estimation. DSLM is a global optimization version extended from Levenberg-Marquardt (LM) algorithm, which is a gradient-based local optimization algorithm.

### Least squares and Levenberg-Marquardt algorithm

The optimal parameters of the proposed dynamic systems are typically estimated by minimizing the sum of errors between observation data set and the mathematical model. A least squares method is the standard approximate solution for parameter estimation by minimizing the sum of squares of the errors between the observation data set and estimated variables of the model function. A data set consists of *n* time-series points (*t*_*i*_, ${\tilde {y}}_{i})$, *i*=1,…,*n*, where *t*_*i*_ is observation time and $\tilde {y}_{i}$ is an observation. Suppose that the upper (**u**) and lower (**l**) boundary of the parameters **x** are given. The constrained optimization problem can be defined by the notation of fitting a model to *n* observations using parameter vector of size *p*, given *R*(**x**) which is continuously differentiable,

(9) $$\begin{array}{@{}rcl@{}} \arg\min_{\textbf{x}} && F(\textbf{x}) = {R(\textbf{x})}^{\top}R(\textbf{x}) = \sum_{i=1}^{n}{r_{i}}^{2}, \\ && \text{s.t.}\indent l_{j} \leq x_{j} \leq u_{j}, 1 \leq j \leq p,  \end{array} $$

where $r_{i} = \tilde {y}_{i} - f(t_{i}, \textbf {x})$ and *f*(*t*_*i*_,**x**) is the model function that has *p* parameters. Here, the goal is to find optimal **x**^∗^ subject to minimization of the function *F*(**x**).

In order to solve the non-linear least squares problem, Isaac Newton proposed Newton’s method to find the minimum or maximum of a function, *F*(**x**), using the first (Jacobian) and second (Hessian)-order partial derivatives of the function [18]. Newton’s method can converge quickly if the initial guess is close to the optimum. However, it is often difficult or impossible to obtain the Hessian matrix of the object function. The steepest gradient method is a first-order optimization algorithm using gradient descent to find a local minimum or maximum [19]. It finds an optimum quickly even though the initial guess is far from the optimum and the system size is very large. However, as it goes close to optimum, it is often infeasible due to the constant step size. The Gauss-Newton algorithm remedies the shortcomings of both Newton method and the steepest gradient method. The Gauss-Newton method has an advantage that it does not need the second derivatives, and it has quadratic final convergence if initial guess is close to the optimum. Generally, if the function has small curve, it is expected to be super convergence. However, it may not produce a good performance if the curve of the first derivative of the function varies slowly, since it ignores the nonlinearity part of Hessian matrix.

To improve these limitations, the Levenberg-Marquardt algorithm (LM) is proposed by Levenberg and Marquardt [20–22]. LM algorithm defines,

(10) $$ ({J_{f}(x)}^{\top}J_{f}(x) + \mu I)\triangle x = {J_{f}(x)}^{\top}R(x)  $$

where *J*_*f*_(*x*) is the first differential function of *f*(*x*), *μ* is a positive scalar called a Marquardt damping parameter, and *I* is an identity matrix. LM can be viewed as a blend of the Gauss-Newton method and the steepest descent method. LM shows similar performance as the Gauss-Newton method if *μ* is small, while it behaves like the steepest descent method when *μ* is large. If *μ* is zero, it will be exactly the Gauss-Newton method. Hence, LM is an adaptive algorithm to retain strength of the steepest gradient method, the Newton method, and the Gauss-Newton method. However, although LM is robust to find an optimal minimum, it has a limitation of local optimization. Therefore, it may fail to find the global minimum if it starts with an initial value belonging to other local curves, which is not suitable to solve the non-linear problems in many cases due to non-convexity.

### Discrete Selection Levenberg-Marquardt (DSLM)

**Fig. 7:**
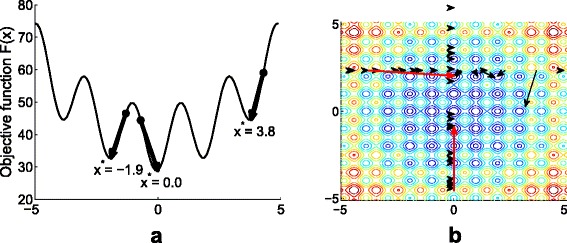
Convergence on DSLM. **a** LM algorithm quickly converges to local minima from the initials, not a global one. In this objective function, there are multiple local minima (when *x*=±3.8,±1.9, and 0). **b** Illustration of the process that DSLM finds the global optimum on iterations, where the red arrows show the update of each variable after checking local optima (*black arrows*)

Our proposed Discrete Selection Levenberg-Marquardt (DSLM) is motivated by the following observation. Although the LM method converges quickly to an optimum solution, it tends to fall into the local optimum rather than a global solution. Figure 7a illustrates that the LM algorithm can converge to different local minima with different initial values. Along with the direction of the initials, LM converges to the local optima on the curvature. Note that the objective function *F*(**x**) in (9) is non-convex, in which there are multiple local optima. Despite the lack, if it is possible to discretize the space to cover every possible local optima, LM may be extended to a global optimal searching method.

A naive global optimization is a NP-hard problem due to the curse of searching dimensions. Hence, most of global optimizations such as genetic algorithm and simulated annealing have adapted heuristic approaches [23–25]. DSLM seeks to search the global optimum for each dimension of **x** iteratively, instead of the entire *p* dimensions of **x** which causes of the infeasibility. This concept reduces the searching space to linear while in the mean time preserving the high performance as a local optimization does. DSLM mainly includes two procedures, selection and discretization.

#### Selection

Like other non-linear least squares algorithms, DSLM starts with initial values **x**^0^ for iterative computation. To avoid the local optima, DSLM attempts to vary the initial guesses iteratively within the dimension of each parameter by fixing other parameters. Denote *S*_*i*_ as a searching space corresponding to parameter *x*_*i*_. Global optimizers search the comprehensive spaces with complexity of *S*_1_×*S*_2_×⋯×*S*_*p*_ for one iteration. On the other hand, the complexity of searching space in DSLM becomes *S*_1_+*S*_2_+⋯+*S*_*p*_, because it iteratively searches each parameter space. Then, it compares the scores of the functions calculated with an updated **x** by conducting the LM method and uses the output as the new initial values. Figure 7b gives an example illustrating the selection of parameters in a 2-dimension space. The arrows show the direction of function scores by varying values on the horizontal axis with a fixed value on the vertical axis. The score of the function is calculated from the result to which the LM algorithm converges. Once the optimal value is calculated for one specific dimension, the parameter is updated as the new parameter. Then, the algorithm seeks the next optimal parameter with fixed parameters previously chosen as the optimum. The selection process iterates until all parameters converge.

#### Discretization

Discretization of each parameter space is the key part which will affect the performance and computational cost for DSLM algorithm. Given *N* number of discrete spaces of a parameter, the discrete spaces can be denoted as a vector, *D*=(*D*^1^,…,*D*^*N*^). However, the function score is not necessarily to be calculated for all discrete spaces. The LM method updates the initial ${x_{k}^{0}}$ to the new parameter, ${x}_{k}^{new}$ subject to converging toward its local optimum. Then, the DSLM algorithm marks the spaces between *D*^*k*^ and $D^{k^{'}}$ where ${x_{k}^{0}}$ and $x_{k}^{new}$ belong to, respectively. It further reduces the space to check. Unlike other competing algorithms using discretization, DSLM’s discretization does not affect the accuracy of the solution but only for preventing rechecking the space.

#### Pseudo-code

The following pseudo-codes, Algorithms 1 and 2, briefly illustrate the DSLM algorithm. DSLM starts with randomly chosen initial values **x**^0^={*x*_1_,…,*x*_*p*_}. It iterates updating **x** until **x** converges or the number of iterations is bigger than a maximum constant. DSLM ensures *F*(**x**^′^)≤*F*(**x**), where *F*(**x**) is the function score with **x**. Lines 5−12 of Algorithm 2 illustrate the optimum selection for each parameter and marking the discrete spaces. After the selection of all parameters, it conducts the LM algorithm to obtain its comprehensive local optimum. The LM method was implemented as the way George, Sam and Ting proposed [26].





## Discussion

### Algorithm assessment

We assessed the performance of DSLM with classical multidimensional benchmark functions and the phagocyte transmigration system, while comparing to global optimizers such as the genetic algorithm (GA) and the stimulated annealing algorithm (SA). Being the most widely-used benchmark functions for global optimization test, the Rastrigin function and the Michalewics function of various dimensions (*d*) were computed (Table 2). The Rastrigin function has several local minima, but the minima are regularly distributed. The true minimum scores and the optimal parameters are both zero for any number of dimensions, and the search domain is defined as −5.12≤*x*_*i*_≤5.12(1≤*i*≤*d*). The Michalewics function has *d*! local minima, where the parameter for the steepness of the valleys and ridges (*m*) is given as 10. The true solution of the Michalewics function is known as −1.8013 when *d*=2, −4.687658 when *d*=5, and −9.66015 when *d*=10. The global minimum of the 10-dimensional Michalewics function occurs when *x*={2.2,1.57,1.29,1.92,1.72,1.57,1.45,1.76,1.66,1.57}. The search domain is given as 0≤*x*_*i*_≤*π*,1≤*i*≤*d*.

**Table 2:** Benchmark functions

Name	Function (*F*(*x*))	Dimension (*d*)
Rastrigin	$10d + \sum _{i=1}^{d} [{{x_{i}^{2}}-10\cos (2\pi x_{i})}]$	*d*={2,5,50}
Michalewics	$-\sum _{i=1}^{d} [\sin (x_{i})\sin ^{2m}(i{x_{i}^{2}} / \pi)]$	*d*={2,5,10}

**Fig. 8:**
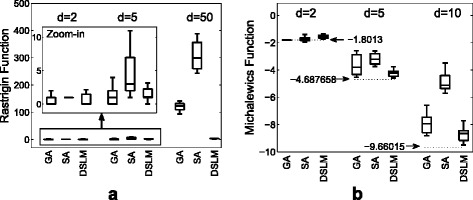
Comparison of the global optimizers. **a** Rastrigin and (**b**) Michalewics

We conducted the assessment with two, five, and 50-dimensional Rastrigin functions and two, five, and ten-dimensional Michalewics functions, by performing GA, SA, and DSLM ten times for each with the randomly generated initial guesses. For the experiments, the three algorithms were all implemented in MATLAB R2012a, where ‘*ga*’ and ‘*simulannealbnd*’ functions that Global Optimization Toolbox provides were used for GA and SA respectively. Although all global optimizers showed the good performances evenly (*F*(*x*)^∗^≈0) when *d*=2, the outstanding achievement of DSLM was appeared for both Rastrigin and Michalewics functions when the dimension increased (e.g., *d*=50) (Fig. 8). The optima of DSLM were 3.50±0.96 and −8.69±0.58 for 50-dimensional Rastrigin function and ten-dimensional Michalewics function, while them of GA and SA were 126.63±33.34 and 303.48±53.03 for Rastrigin and −7.88±0.70 and −4.86±0.68 for Michalewics. Note that the true optima of any dimensional Rastrigin and ten-dimensional Michalewics functions are 0 and −9.66015 respectively.

Moreover, we empirically examined the number of the iterations that the optimizers need to converge. We considered the number of generation for GA and iteration numbers for SA and DSLM. As described in Table 3, DSLM had least iterations among them as well as the slowest increase when the dimension increases.

**Table 3:** Performance comparison (iteration numbers)

		d=2 (mean ±std)	d=5 (mean ±std)	d=50 (mean ±std)
Rast.	GA	51 ± 0	70.1 ± 14.41	174.9 ± 62.7
	SA	1744.4 ± 410.84	5003.5 ± 2126	41921 ± 6326.1
	DSLM	5.2 ± 2.44	4.6 ± 1.64	22.5 ± 4.32
Mich.	GA	51 ± 0	58.3 ± 8.98	81.6 ± 29.6
	SA	1760.3 ± 765	3606.3 ± 880.7	8956.3 ± 2171.3
	DSLM	3.5 ± 1.08	4.9 ± 1.6	7.4 ± 3.56

**Fig. 9:**
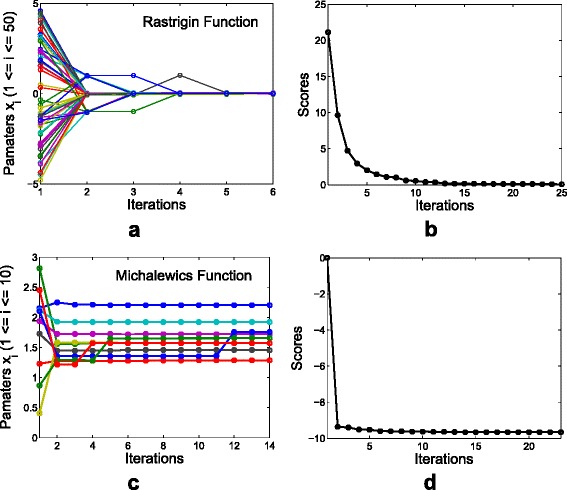
Iterations of the optimization. **a** illustrates the trajectories of the parameter updates on the iterations and (**b**) shows the score convergence of DSLM for the Rastrigin function. **c** and (**d**) are for the Michalewics function. The minimum scores of the Rastrigin function and the Michalewics function are known as zero for all of dimensional spaces, and −9.66015 for 10-dimensional space, respectively

The convergent procedure of DSLM for both 50-dimensional Rastrigin function and 10-dimensional Michalewics function are illustrated in Fig. 9. The 50 parameters of the Rastrigin function converged to zero after five iteration as shown in Fig. 9a. The scores that monotonically convergent on the iterations are observed in Fig. 9b. The trajectories of both the parameter updates and the score convergence for the Michalewics function are shown in Fig. 9c, d.

**Fig. 10:**
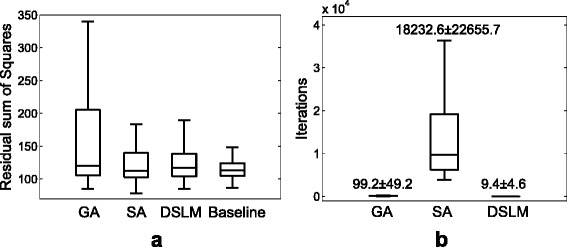
The assessment of the performance on the phagocyte transmigration model. *Baseline* is the residual sum of squares. SA and DSLM show similar performance of less mean and standard deviation than GA, whereas DSLM needed much less iteration numbers than SA

We also tested the global optimizers with the phagocyte transmigration model in order to assess the performances on the biological system. With the residual histamine model equations, (2) and (3), the ground truth parameters and the simulation data with white noise (*σ*=0.1) were randomly generated for the test. Then, the minimized scores and iteration number are examined and compared by repeating 100 times (Fig. 10). In Fig. 10a, *baseline* shows the residual sum of squares with the ground truths, which is the minimum errors that the simulated data intrinsically has. SA and DSLM show similar performance of less mean and standard deviation than GA, whereas DSLM needed much less iteration numbers than SA in Fig. 10b.

## Conclusions

In this paper we presented a computational modeling framework for the study of biological system in response to biomedical implants. As an example of application, phagocyte transmigration has been studied to tackle the problem of fibrotic tissue formation surrounding the biomaterial implants, which causes implantation failure.

Foreign body reactions are complicated processes. Many cells and cellular products participate in the processes. To model such complex reactions, we focused on the key components identified in many previous works. Furthermore, the proposed model system is aimed at modeling short-term acute inflammatory responses at which there are many similarities between wound healing responses and foreign body reactions, while most of the chronic diseases, including foreign body reactions and fibrosis, are the long term outcomes. Based on the in-depth understanding of the successive reactions, mathematical equations are developed.

To complete the quantitative modeling, reverse engineering is conducted to estimate the parameters of the mathematical modeling equations. We developed a global optimization technique, DSLM, which overcomes the limitations of existing local optimization algorithms such as the LM algorithm and shows better performance than other global heuristic approaches. The proposed DSLM globally estimates the optimal parameters of the phagocyte transmigration modeling system, which discloses the inner nature of the dynamic system. To verify the global optimization capability of the modeling, the system was tested with the Rastrigin function and the Michalewics function. Nevertheless, DSLM is rather optimized to this study not as a general optimizer yet, since it needs more concrete analysis theoretically and empirically as a future work. With mathematical equations and estimated parameters by DSLM, we are able to further simulate and predict different aspects of phagocyte transmigration process under different conditions of system inputs.

## Ethics statement

This research does not involve human subjects, human materials or human data. All animal data came from an early publication (Tang et al., PNAS, 95:8841-6, 1998). The animal experiments for that study were approved by the Albany Medical College’s Animal Care and Use Committee (IACUC) and in accordance with the National Institutes of Health guidelines for the use of laboratory animals.

## Availability of supporting data

The generated transmigration data named Transmigration.xls and MATLAB source codes DSLM.zip are publicly accessible at: http://faculty.tamuc.edu/mkang/DSLM. The measurements of transmigration data can be found in Additional file 1. The source code as well as the synthetic data are provided in Additional file 2.

## Additional files

Additional file 1
**List of histamine, P selectin, PMN and M** ***Φ*** **measurement data named.** (XLS 31 kb)

Additional file 2
**Details of ordinary differential equation solution and synthetic data generation.** (PDF 149 kb)
