# Supplementary material for: Computational modeling of phagocyte transmigration for foreign body responses to subcutaneous biomaterial implants in mice
Source: BMC Bioinformatics. 2016 Feb 29;17:111. doi: 10.1186/s12859-016-0947-3 (PMC4772519; doi:10.1186/s12859-016-0947-3)
Supplement: Additional file 2 — Details of ordinary differential equation solution and synthetic data generation. (PDF 149 kb) [file 12859_2016_947_MOESM2_ESM.pdf]

Computational modeling of phagocyte transmigration for foreign body responses to subcutaneous biomaterial implants in mice

Mingon Kang, Liping Tang, and Jean Gao

1 Discrete Equations

Differential equations are commonly used as mathematical modeling method to represent dynamic evolution of biological propagation. However, differential equations is difficult to compute accurate simulation and apply algorithms. Therefore, the most popular way is to convert differential equations to discrete equations of the following type:

$$x_{n+1} = f(x_n, x_{n-1}, \ldots), x_0 = a_0. \tag{1}$$

A discrete equation needs an initial value for the first state, and it iterates computing next states recursively depending on previous state. Basically, the differential equation form can be represented in discrete-time with constant sampling interval $\triangle$t as:

$$\dot{y} = ay + bu(t), \tag{2}$$

$$y_{k+1} = \Phi y_k + \Gamma u_k, \tag{3}$$

where the integer constant k is the sample index, and

$$\Phi = e^{a\triangle t}, \tag{4}$$

$$\Gamma = \int_0^{\triangle t} be^{at}dt = \frac{b}{a}(e^{a\triangle t} - 1). \tag{5}$$

In this way, mathematical differential equations for the consecutive events during transmigration can be transformed to the following equations. For residual histamine,

$$C_{rh(k+1)} = e^{-k_{rhch}\triangle t}C_{rh(k)} - \frac{1}{k_{rhch}}(e^{-k_{rhch}\triangle t} - 1)U_{xrmc(k)}. \tag{6}$$

For histamine,

$$C_{h(k+1)} = e^{-k_{hs}\triangle t}C_{h(k)} - \frac{1}{k_{hs}}(e^{-k_{hs}\triangle t} - 1)k_{rhch}C_{rh(k)}I_{mc}(t). \tag{7}$$

For histamine receptors,

$$C_{hr(k+1)} = e^{-k_{rhs}\triangle t}C_{hr(k)} - \frac{1}{k_{rhs}}(e^{-k_{rhs}\triangle t} - 1)\frac{k_{hchrt}C_{h(k)}}{k_{hchrb} + C_{h(k)}}. \tag{8}$$

For P/E selectins,

$$C_{s(k+1)} = e^{-k_{ss}\triangle t}C_{s(k)} - \frac{1}{k_{ss}}(e^{-k_{ss}\triangle t} - 1)\frac{k_{hcst}C_{h(k)}}{k_{hcsb} + C_{h(k)}}. \tag{9}$$

For permeability of PMN,

$$C_{pmnp(k+1)} = e^{-k_{pmnps}\triangle t} C_{pmnp(k)} - \frac{1}{k_{pmnps}}(e^{-k_{pmnps}\triangle t} - 1)\frac{k_{pmnipt} C_{h(k)} C_{s(k)} I_{pmns}(t) I_{pmnhr}(t)}{k_{pmnipb} + C_{h(k)} C_{s(k)} I_{pmns}(t) I_{pmnhr}(t)}. \tag{10}$$

For PMN

$$C_{pmn(k+1)} = e^{-k_{pmns}\triangle t} C_{pmn(k)} - \frac{1}{k_{pmns}}(e^{-k_{pmns}\triangle t} - 1) C_{pmnp(k)}. \tag{11}$$

For permeability of MΦ

$$C_{mpp(k+1)} = e^{-k_{mpps}\triangle t} C_{mpp(k)} - \frac{1}{k_{mpps}}(e^{-k_{mpps}\triangle t} - 1)\frac{k_{mpipt} C_{h(k)} C_{s(k)} I_{mps}(t) I_{mphr}(t)}{k_{mpipb} + C_{h(k)} C_{s(k)} I_{mps}(t) I_{mphr}(t)}. \tag{12}$$

For MΦ

$$C_{mp(k+1)} = e^{-k_{mps}\triangle t} C_{mp(k)} - \frac{1}{k_{mps}}(e^{-k_{mps}\triangle t} - 1) C_{mpp(k)}. \tag{13}$$

The initial value of Equation (6) was set by the mean of the variables of observation data set at time 0, and initial values for other Equations (7), (8), (9), (10), (11), (12), (13) were set as 0 for simplification.

2 Synthetic Data Using Iterative Weighted Mean Algorithm

The small sample size makes it hard to estimate parameters of the system without bias. To overcome the shortage of limited available experiment data, we generated synthetic data. By generating synthetic data, we can have not only supplementary data to provide more reasonable result, but also consistent data with naturally removed outliers.

To generate the synthetic data from small size real biological data, iterative weighted mean algorithm (IWM) was used. While arithmetic mean assumes same weight for each variable, IWM calculates the mean using adaptive weight, $\mu_k = \sum_{i=1}^{n} w_i x_i / \sum_{i=1}^{n} w_i$, where $w_i = e^{-(x_i - \mu_{k-1})^2/\varsigma^2}$. Here, normal distribution with previous mean, μ_k and variance, σ^2 at the iteration k, is used to determine the weight. The algorithm iterates until the weight difference, w_k - w_{k-1}, is smaller than a sufficiently small constant ϵ. The initial mean starts with an arithmetic mean, and is updated to a more precise value considering different observation weights. After the calculation

of the mean vector, the synthetic data is generated by normal distribution with the finally calculated mean by the iterative weighted mean algorithm and the variance of original data to mimic as close as possible the original data dispersion. Then, the synthetic data was linearly interpolated due to the sparse time-series (two-hour interval).
